# Analysis of the Performance and Accuracy of a PSA and PSA Ratio-Based Nomogram to Predict the Probability of Prostate Cancer in a Cohort of Patients with PIRADS 3 Findings at Multiparametric Magnetic Resonance Imaging

**DOI:** 10.3390/cancers16173084

**Published:** 2024-09-05

**Authors:** Franco Palmisano, Vito Lorusso, Rebecca Legnani, Vincenzo Martorello, Carlotta Nedbal, Pietro Tramanzoli, Federica Marchesotti, Simona Ferraro, Michele Talso, Antonio Maria Granata, Maria Chiara Sighinolfi, Bernardo Rocco, Andrea Gregori

**Affiliations:** 1 Department of Urology, ASST Fatebenefratelli-Sacco Hospital, 20157 Milan, Italy; rebecca.legnani@studenti.unimi.it (R.L.); vincenzo.martorello@asst-fbf-sacco.it (V.M.); nedbal.carlotta@asst-fbf-sacco.it (C.N.); tramanzoli.pietro@asst-fbf-sacco.it (P.T.); marchesotti.federica@asst-fbf-sacco.it (F.M.); michele.talso@asst-fbf-sacco.it (M.T.); antonio.granata@asst-fbf-sacco.it (A.M.G.); andrea.gregori@asst-fbf-sacco.it (A.G.); 2 Pediatric Department, Buzzi Children’s Hospital, 20154 Milan, Italy; simona.ferraro@asst-fbf-sacco.it; 3 Department of Urology, ASST Santi Paolo e Carlo, University of Milan, 20142 Milan, Italy; mariachiara.sighinolfi@asst-santipaolocarlo.it (M.C.S.); bernardo.rocco@unimi.it (B.R.); 4 University of Milan, 20122 Milan, Italy

**Keywords:** prostate cancer, MRI, PIRADS 3, nomogram

## Abstract

**Simple Summary:**

Prostate cancer (PCa) detection and risk stratification in patients with PIRADS 3 represents a challenge, since it depicts a clinical scenario in which the chance of unnecessary biopsy procedures and overdiagnosis is still high. For these reasons, in this study, we analyzed and evaluated the performance of a nomogram previously developed by our group and based on age, total PSA and PSA ratio to estimate the probability of harboring PCa and help with discriminating both PCa with ISUP < 3 and ISUP ≥ 3 in patients scheduled for prostate biopsy at our institution and with a recent finding of PIRADS 3 at multiparametric MRI (mpMRI).

**Abstract:**

Background: PIRADS score 3 represents a challenge in prostate cancer (PCa) detection with MRI. Our study aimed to evaluate the application of a nomogram on a cohort of patients with PIRADS 3. Methods: We analyzed 286 patients undergoing fusion prostate biopsy from January 2020 to February 2024. Only PIRADS 3 patients were included. Two nomograms, previously developed and based on clinical variables such as age, total PSA (specifically 2–10 ng/mL) and PSA ratio were applied to estimate the probability (Nomograms A and B) for PCa Grade Group (GG) > 3 and GG < 3. Results: Out of the 70 patients available for analysis, 14/70 patients (20%) had PCa, 4/14 were GG 1 (28.6%), 1/14 were GG 2 (7.1%), 5/14 were GG 3 (35.8%), 2/14 were GG 4 (14.3%) and 2/14 were GG 5 (14.3%). The median probability of PCa GG > 3 and GG < 3 was 5% and 33%, respectively. A significant difference (*p* = 0.033) was found between patients with negative versus positive biopsy for Nomogram B. There was a significant difference (*p* = 0.029) for Nomogram B comparing patients with GG < 3 and GG > 3. Using a cut-off of 40% for Nomogram B, sensitivity and specificity were 70% and 80%, respectively. Conclusions: This cohort has a low probability of harboring PCa especially ISUP > 3. Nomogram B has good accuracy for discriminating patients with PCa from those with negative biopsy.

## 1. Introduction

Prostate cancer (PCa) is one of the most common cancers in men, and early detection is crucial for effective management [1]. Prostate-specific antigen (PSA) testing has been widely used as a screening tool for prostate cancer, but its accuracy has been the subject of ongoing debate [2]. While PSA is a useful marker, it has limitations in differentiating between benign and malignant prostate conditions, leading to both the over-diagnosis and under-diagnosis of clinically significant disease [3,4]. Accurate prediction of disease behavior is critical, because radical treatment is associated with high morbidity [3]. One promising approach to improve the accuracy of PCa detection is the use of Multiparametric Magnetic Resonance Imaging (mpMRI) of the prostate, which has shown promising results in identifying clinically significant PCa [4,5]. In fact, with the introduction of mpMRI in clinical practice, several studies and trials have demonstrated an improvement in PCa detection and a reduction in the overdiagnosis phenomenon [6,7,8,9,10]. The Prostate Imaging Reporting and Data System (PIRADS), currently at its version 2.1 [11], is widely employed to standardize the interpretation of mpMRI findings, categorizing lesions on a scale from 1 to 5 based on their likelihood of being clinically significant PCa. Among these categories, PIRADS 3 lesions represent a particular diagnostic challenge. These lesions are classified as having an intermediate probability of malignancy with studies reporting different cancer detection rates ranging between 21% and 16% [12]. The uncertainty associated with PIRADS 3 lesions complicates clinical decision making, as it is unclear whether to proceed with biopsy, active surveillance, or other management strategies. This uncertainty is given by the fact that PIRADS 3 lesions often yield inconclusive or non-definitive results at biopsy, leading to the potential for both over-diagnosis and under-diagnosis of clinically significant PCa. In response to this challenge, an increasing need has been raised to develop additional tools and methods to stratify better the risk associated with PIRADS 3 findings, thereby enhancing diagnostic accuracy and patient management [12,13]. Among the proposed tools, advancements have been made with the implementation of Prostate-Specific Membrane Antigen (PSMA)–Positron Emission Tomography (PET)-based imaging and targeting; however, mpMRI remains the cornerstone for the diagnosis of PCa [14,15].

In this study, we aimed to evaluate the use and accuracy of a PSA and PSA ratio-based nomogram for predicting the probability of PCa in a cohort of patients with PIRADS 3 findings on multiparametric MRI.

## 2. Materials and Methods

We retrospectively analyzed data from 286 patients who underwent, at our institution, mpMRI of the prostate followed by targeted biopsy between January 2020 and March 2024. Patients were eligible for inclusion if they had a Prostate-Specific Antigen (PSA) level between 2 and 10 ng/mL and one or more PIRADS 3 lesions on mpMRI. Patients with a history of prior prostate biopsy or treatment for PCa were excluded. Patients with PIRADS > 3 were equally excluded. 

Demographic and clinical data were collected for all patients, including age, total PSA, free PSA, PSA ratio, and PSA density. MpMRI findings, including prostate volume, number of PIRADS 3 lesions, lesion diameter, and lesion location, were also recorded.

Total and free PSA were measured by one assay (Roche), since a large body of literature has shown that PSA assays, in particular for free PSA, are poorly harmonized, and mixing results obtained by different assays may introduce a bias in patients’ classification [16].

All patients underwent targeted biopsy of the PIRADS 3 lesions identified on mpMRI with additional random systematic biopsies performed as well. Pathological analysis was conducted by an expert genitourinary pathologist, who determined the Gleason score and International Society of Urologic Pathologists (ISUP) Grade Group for both the targeted and random biopsies.

All prostate biopsies were performed using a transrectal ultrasound-guided fusion technique with the aid of software for the superimposition of the multiparametric MRI images of the prostate. For each biopsy procedure, 4 samples were taken from the lesions identified on the MRI, which was followed by a standard 12-core random prostate sampling. All the biopsies were performed by two surgeons specifically trained in fusion prostate biopsy and mpMRI reading, and the patients’ prostate MRI scans were reported and/or reviewed by an expert uroradiologist before the biopsy procedure.

**Figure 1:**
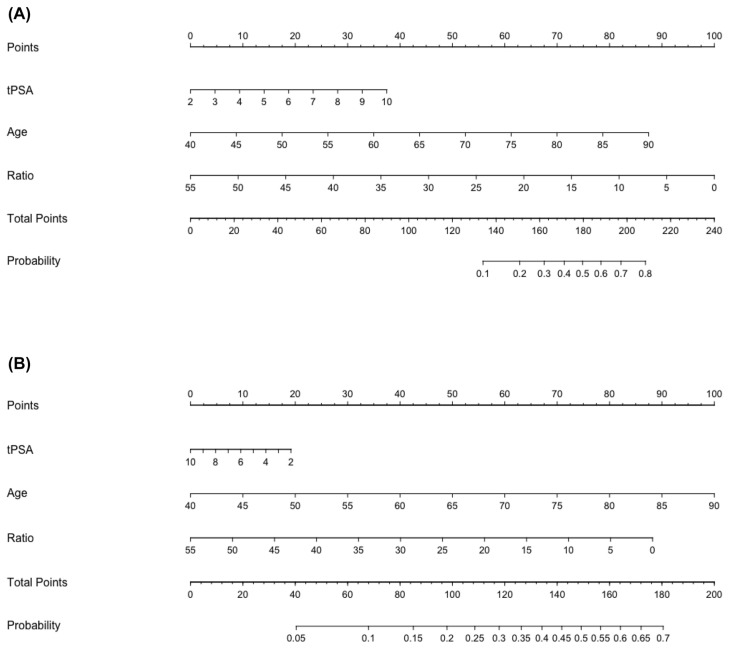
Nomograms used to estimate the probability of ISUP ≥ 3 (**A**) and ISUP < 3 (**B**) prostate cancer in patients with PIRADS 3 lesions (reproduced with permission from Ferraro et al. [17]).

An institutional developed nomogram (Figure 1), described elsewhere [17], was used to estimate the individual risk of high-grade (ISUP > 3) and low-grade (ISUP < 3) PCa for each patient based on age, total PSA and PSA ratio. Specifically, as described in the previous study by our group [16], the nomograms were conceived in such a way: Nomogram A was designed to predict the risk of advanced prostate cancer (ISUP grade ≥ 3), helping in differentiating between advanced PCa and ISUP < 3 PCa or no cancer cases. Nomogram B was developed to discriminate between ISUP < 3 PCa and no cancer cases. Moreover, the Area Under the Curve (AUC) was 0.80 and 0.63 for Nomograms A and B, respectively. Regarding the calibration, Nomogram A seemed to underestimate the risk when the estimated probability was higher than 30%, whilst Nomogram B was shown to overestimate the risk of PCa when the calculated probability was greater than 50%. Taking into account the Hosmer–Lemeshow Goodness-of-Fit test, both Nomograms A and B showed good fitness with a reported *p*-value of 0.35 and 0.62, respectively. Summarizing, Nomogram A demonstrated to be reliable and particularly useful for identifying patients at risk of advanced PCa. Meanwhile, Nomogram B seemed to perform less well, but it could still be useful in specific clinical context cases, such as patients with PIRADS 3 findings [17].

Categorical variables were reported with numbers and percentages, while continuous variables were reported with median and interquartile range (25th–75th percentile). Descriptive statistics were used to summarize and analyze the patient characteristics and biopsy results such as the pre-biopsy variables, the data of the MRI, and the final pathologic report. Univariate comparative statistical analysis was performed using the Mann–Whitney U test for continuous variables. Discrete variables were compared using Chi-squared (χ^2^) analysis. Statistical significance was set at *p* < 0.05. The Mann–Whitney U test and the chi-square test were used for statistical analysis with a *p*-value < 0.05 considered statistically significant. Receiver Operating Characteristic (ROC) curve analysis was used to evaluate the Area Under the Curve (AUC) of the nomograms. All statistical analyses were performed using SPSS version 29.0 (SPSS Inc., Chicago, IL, USA).

## 3. Results

The clinical and demographic characteristics of the patients are summarized in Table 1.

The median age of the patients at the time of biopsy was 66 years (range: 61–73). The median PSA value was 5.50 ng/mL (IQR: 3.9–6.9 ng/mL), while the PSA ratio was 17% (IQR: 12–23%). The median PSA density, on the other hand, was 0.10 ng/mL/mL (IQR: 0.06–0.12 ng/mL/mL). The median prostate volume was 63 cc (IQR: 49–100 cc). Overall, 77.1% of the patients (54/70) had a single PIRADS = 3 lesion, while the remaining 22.9% (16/70) had a maximum of two PIRADS = 3 lesions. The median diameter of the lesions was 8 mm (IQR: 6–10.5 mm). A positive biopsy was found in 20% of the patients (14/70), specifically 28.6% (4/14) were ISUP GG 1, 7.1% (1/14) was ISUP GG 2, 35.8% (5/14) were ISUP GG 3, 14.3% (2/14) were ISUP GG 4 and 14.3% (2/14) were ISUP GG 5. Data regarding the estimation of PCa risk according to the nomogram are shown in Table 2. 

**Figure 2:**
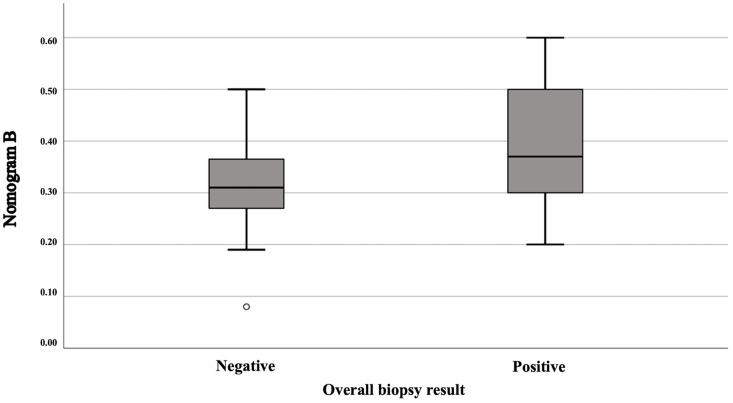
Box plot showing the difference in the estimated probability according to Nomogram B between patients with positive and negative biopsy results.

Applying our nomogram, as expected, the median probability of finding a PCa ISUP GG ≥ 3 (Nomogram A) was 5% (IQR: 0–15%), while for a PCa ISUP GG < 3 (Nomogram B), it was 33% (IQR: 27.5–40%). Looking in more detail, for patients with a positive biopsy, the median values of Nomogram A and Nomogram B were 8% (IQR: 0–20%) and 37% (IQR: 29.5–50.75%) respectively; for patients with a negative biopsy, on the other hand, the median was 5% (IQR: 2–13%) for Nomogram A and 31% (IQR: 27–36.75%) for Nomogram B. This last probability calculated with Nomogram B was statistically different (Figure 2) in patients with a positive biopsy compared to patients with a negative biopsy (*p* = 0.033), while no statistically significant difference was found for the probability calculated with Nomogram A in the two groups (*p* = 0.74). 

**Figure 3:**
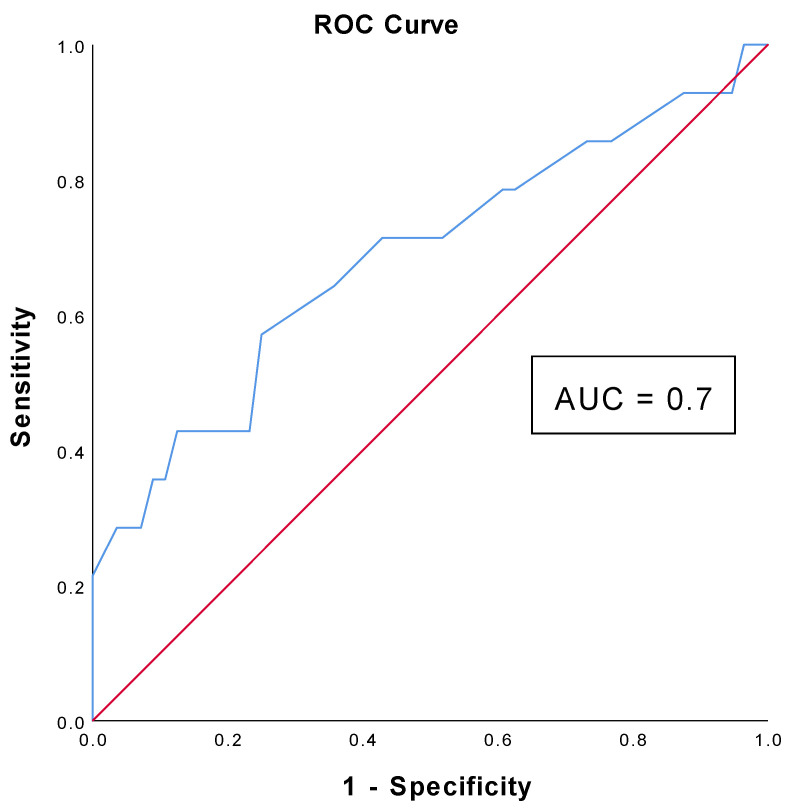
Diagram showing the Receiver Operating Characteristic (ROC) curve analysis and the Area Under the Curve (AUC) value of Nomogram B.

Regarding Nomogram B, a significant difference was also found when comparing patients with ISUP < 3 and with ISUP ≥ 3 (*p* = 0.029). The AUC of Nomogram B is equal to 0.685 (CI: 0.511–0.858) and using a cut-off of 40%, the nomogram shows a sensitivity of 70% and a specificity of 80% (Figure 3). Moreover, using this cut-off for Nomogram B, around 63% (44/70) of the patients would have been spared an unnecessary biopsy procedure without missing any cancer.

In patients with PSA density > 0.15 (5/14 patients with positive biopsy), the median values of Nomogram A and Nomogram B were 18% (IQR: 0–29%) and 35% (IQR: 26.50–54.50%) respectively. 

## 4. Discussion

The cohort of patients examined in this study shows a prevalence of positive cases for PCa of 20%, whilst the negative cases are 80%; these data are consistent with the results of other studies in which the incidence of PCa was analyzed based on the categories of the PIRADS v2.1 score [18]. Some studies have reported cancer detection rates ranging from 15% to 31% for PIRADS 3 category [19,20]. For example, a study by Schoots et al. reported a cancer detection rate of approximately 22% for PIRADS 3 lesions, suggesting that while these lesions carry a moderate risk, the majority do not represent clinically significant disease [21]. Moreover, Thompson et al. found that most cancers detected in PIRADS 3 lesions were low grade (GG 1 or 2) with fewer cases of clinically significant cancer (GG ≥ 3) [22]. This study demonstrates how the use of the nomograms we evaluated, particularly for Nomogram B, can discriminate with significant accuracy patients with positive and negative biopsy results as well as between GG < 3 and GG > 3 cancers. The evaluation with this nomogram could therefore allow us to better evaluate patients in whom a prostate biopsy is truly indicated to confirm the diagnosis of PCa and avoid unnecessary procedures. This finding is supported by similar studies that have explored the application of nomograms and other predictive models in prostate cancer risk assessment [23,24]. As known, the PIRADS 3 category represents a clinical challenge in the interpretation of mpMRI results, for which several studies have proposed the use of different clinical variables and nomograms to guide the clinician in the diagnostic pathway of these patients [25]. In the context of assessing the risk of PCa, one of the most widely used clinical parameters is the PSA density, as also proposed in the European Association of Urology (EAU) guidelines [26]. According to the EAU guidelines, in the presence of a PIRADS 3 lesion and with a PSA density value higher than 0.15 ng/mL/mL, the probability of diagnosing a clinically significant PCa (ISUP > 2) is equal to 30%. This threshold of probability, therefore, suggests the need to perform a prostate biopsy. Using our nomogram, in patients with this cut-off of PSA density and therefore diagnosed with PCa, the probability of identifying a high-grade PCa was 18%, while for a low-grade tumor, it was 35%: these results indicate that our nomogram could be able not only to help in selecting patients who should undergo a biopsy but also to stratify the aggressiveness of cancer itself.

Historically, the European Randomized Study for Screening of Prostate Cancer (ERSPC) risk calculator was the first nomogram designed to predict the risk of prostate cancer after a prostate biopsy. The nomogram used the Prostate-Specific Antigen (PSA) level, the findings of a digital rectal examination (DRE), and the prostate volume to predict the probability of prostate cancer after a systematic biopsy [27]. Later, Thompson et al. developed a separate nomogram based on men who participated in the Prostate Cancer Prevention Trial (PCPT). In the latter, the variables included in this nomogram were total PSA, free PSA, DRE, a family history of PCa, and African American race, accounting also for the subsequent updates [28]. A more recent nomogram was implemented by the Prostate Biopsy Collaborative Group (PBCG). This calculator includes age, PSA, DRE, family history, race, and a prior negative biopsy [29]. The AUC for these models ranges from 0.65 to 0.75, 0.70 to 0.79 and 0.74 to 0.81 for PCPT, ERSPC and PBCG nomograms, respectively [30,31]. However, these nomograms did not focus mainly on a cohort of patients with PIRADS 3; hence, a direct comparison of this aspect is not possible. Nevertheless, as shown in a previous paper [17], the AUC for the nomograms we used was 0.80 and 0.63 for Nomogram A and Nomogram B, respectively. Taking these data into consideration, we believe in this specific cohort of patients with a PIRADS 3 at mpMRI, Nomogram B was found to perform better than Nomogram A.

A study by Mehralivand et al. evaluated the performance of a predictive model that combined clinical variables with mpMRI findings and found that such models could significantly enhance the risk stratification of patients with indeterminate PIRADS scores. Their model showed high predictive accuracy, particularly in identifying clinically significant cancers, which aligns with the current study’s results for Nomogram B. Specifically, including the mpMRI parameters alongside the traditional clinical variables improved the AUC from 64% to 84%. In this study, the authors included 400 patients in the development cohort and 251 patients in the validation cohort and stated that by applying their model, around 38% of biopsies could have been omitted with a 20% risk threshold. However, a specific analysis on PIRADS 3 patients was not performed [32]. 

A 2021 retrospective study led to the development and validation of a nomogram to predict the risk of prostate neoplasia before biopsy in patients with PSA < 20 ng/mL, using parameters such as age, PSA, free PSA, and prostate volume. This approach showed good results in terms of diagnostic accuracy (AUC = 0.857). Using a threshold value of 15%, the sensitivity and specificity of the nomogram were 95.6% and 42.5%, respectively, while the negative predictive value was 93.4%. Using this cut-off, it would have been possible to avoid 25% of biopsies, missing the diagnosis of PCa in only 4.4% of patients [33]. However, it is important to consider that measuring prostate volume requires imaging techniques such as transrectal ultrasound or magnetic resonance imaging, which can involve high costs, invasiveness, and limited availability, potentially leading to delays in patient evaluation and diagnosis [34,35]. 

In our study, the sensitivity and specificity of 70% and 80%, respectively, for Nomogram B at a 40% cut-off are within the range reported in other studies. Previously, Van Leeuwen et al. reported similar metrics in their evaluation of a predictive model for PIRADS 3 lesions, highlighting the potential of nomograms to serve as valuable decision-support tools in clinical practice [36]. Other nomograms, on the other hand, make use of additional biomarkers such as the 4K score, which is based on a kallikrein whose value is correlated with an increased risk of developing prostate cancer. In this retrospective study, the authors included 574 men with only 68 (20%) reported PIRADS 3 at mpMRI. The nomogram used demonstrates high accuracy (AUC = 0.84) in predicting the presence of prostate cancer. It was also estimated that in the overall cohort of patients examined, it was possible to avoid 10% of the biopsies performed without missing the diagnosis of any clinically insignificant tumors and only 1% of clinically significant ones. Compared to our nomograms, the 4K score integrated with mpMRI has similar accuracy to our Nomogram A based on the value of AUC, performing better in high-grade PCa [37]. 

Several other biomarkers have been proposed over the years and integrated with mpMRI to improve diagnostic accuracy and patients’ stratification risk. Among them, ExoDx Prostate Intelliscore (EPI) has been investigated and has shown promising results. Recently, King et al. performed a study on 226 patients who underwent both EPI and mpMRI (with a PIRADS score ≥ 3). They reported that the integration of EPI and mpMRI showed a sensitivity of 96% compared to 91% and 90% for EPI and mpRMI alone, respectively. Moreover, the AUC for EPI and mpMRI combined was 0.8, whilst the values of the AUC for EPI and mpMRI alone were 0.57 and 0.78, respectively. In this study, they also reported that using the integration of EPI and mpMRI, 43% of the patients could have avoided the biopsy procedure [38]. In our study, the percentage of patients that could have been spared a biopsy was found to be around 58% with a cut-off for Nomogram B set at 40% for the risk of PCa. However, the cited study included patients with a PIRADS score ≥ 3 and no data of a subgroup analysis regarding specifically patients with PIRADS 3 only was performed. Thus, such a population compared with our study has a higher pre-test prevalence of harboring PCa. These considerations should be taken into account while analyzing the value of sensitivity. To date, we have found no study exploring these biomarkers and focusing on a population of patients exclusively with PIRADS 3 [39].

Despite the promising results, its clinical use could be compromised by the cost of the test and limited availability [37]. Conversely, the variables included in our nomogram allow the use of easily available clinical parameters and therefore to stratify the patient potentially already at the time of the first clinical consultation without the need for diagnostic imaging investigations. However, our study is not devoid of limitations. Firstly, the number of patients included may be insufficient to ensure a representation of the study population, particularly regarding the number of patients with positive biopsy results. Second, it was an internal validation of the nomograms. However, we decided to include patients from our single center to pay particular attention to minimize the different sources of variability which may be associated with the use of different PSA immunoassays and to patients retrieved from different centers. In fact, for these reasons, we decided to include only patients with total and free PSA measured by one assay (Roche). These decisions also affected the relatively small sample size. Accordingly, this study served as an internal validation, providing important evidence relevant to proceed to an external validation study. Thus, an external validation will be the aim of our future research regarding the application of these nomograms.

## 5. Conclusions

The assessment of PCa risk represents a crucial challenge in clinical practice, especially considering the variety of factors involved in its diagnosis and management.

The results obtained with our nomogram clearly show that the population of patients with a PSA range of 2–10 ng/mL and a PIRADS 3 score have a low probability of developing PCa especially a high-risk tumor with an ISUP score greater than 3.

Furthermore, Nomogram B shows a good ability to discriminate between patients with PCa and those negative for biopsy, indicating a potential supporting role in selecting candidates for prostate biopsy.

Even more significant is the observation that Nomogram B demonstrates excellent accuracy in distinguishing between PCa with ISUP scores of 1–2 and those with ISUP scores greater than 3, thus providing valuable guidance in identifying patients at higher risk of having a more aggressive form of PCa.

The use of such nomograms could therefore facilitate the stratification of PCa risk in this specific category of patients, contributing significantly to the decision-making process regarding the need to undergo prostate biopsy. 

In conclusion, the nomogram used in this study presents itself as a valuable additional tool in the diagnostic repertoire of PCa, offering a more detailed and personalized overview of individual risk and thus contributing to improving the care of patients suffering from this disease.

## Figures and Tables

**Table 1:** Clinical and pathologic characteristics of the patients with PIRADS 3 lesions (*N* = 70).

Variables	Total Number of Patients = 70
**Age**, years [median (range)]	66 (61–73)
**PSA**, ng/mL [median (IQR)]	5.50 (3.9–6.9)
**PSA ratio**, [median (IQR)]	0.89 (0.52–1.3)
**PSA density**, ng/mL/mL [median (IQR)]	0.1 (0.06–0.12)
**Prostate volume**, mL [median (IQR)]	63 (49–100)
**Dimension of the lesion**, mm [median (IQR)]	8 (6–10.5)
**Location of the lesion**, *N* [%]	
- Apex	23 (32.9%)
- Intermediate	26 (37.1%)
- Base	9 (12.9%)
**Gland zone of the lesion**	
- Anterior	13 (18.6%)
- Peripheric	38 (54.3%)
- Transitional	16 (22.9%)
**Overall biopsy result**	
- Negative for PCa	56 (80%)
- Positive for PCa	14 (20%)

IQR (Interquartile range).

**Table 2:** Estimated probability according to Nomogram A (ISUP ≥ 3) and Nomogram B (ISUP < 3).

Variables	Probability [%]
**Nomogram A overall, % [IQR]**	5% (0–15%)
- Patients with positive biopsy	8% (0–20%)
**Nomogram B overall, % [IQR]**	33% (27.75–40%)
- Patients with positive biopsy	37% (29.50–50.75%)

IQR (interquartile range).

## Data Availability

The original contributions presented in the study are included in the article; further inquiries can be directed to the corresponding author.
